# The effect of social relationships on survival in elderly residents of a Southern European community: a cohort study

**DOI:** 10.1186/1471-2318-7-19

**Published:** 2007-08-01

**Authors:** Angel Rodriguez-Laso, Maria Victoria Zunzunegui, Angel Otero

**Affiliations:** 1 Dirección General de Salud Pública y Alimentación, Comunidad de Madrid, Spain; 2 Département de Médecine Sociale et Preventive. Université de Montréal, Canada; 3 Departamento de Medicina Preventiva y Salud Pública, Universidad Autónoma de Madrid, Spain

## Abstract

**Background:**

Comparative evidence regarding the effects of social relationships on mortality in Mediterranean communities will increase our knowledge of their strengths and the ways in which they influence longevity across cultures. Men and women may benefit differently from social relationships because of cultural differences in gender roles. Psychosocial mechanisms such as social support, which may explain the effects of social networks, may also vary by culture.

**Methods:**

Detailed information on the social relationships of a representative sample of 1,174 community-dwelling older adults was collected in Leganés, a city in central Spain. Mortality over a 6-year follow-up period was ascertained. Information on socio-demographic, health and disability variables was also collected. Cox proportional hazards models were fitted separately for men and women and for the combined sample.

**Results:**

Having a confidant was associated with a 25% (95% CI 5–40%) reduction in the mortality risk. The hazard ratio for lack of social participation was 1.5 (95% CI 1.3–1.7). Being engaged in meaningful roles protected against mortality, while receipt of emotional support did not affect survival. These results were comparable for men and women. Having contact with all family ties was associated with reduced mortality only in men. Structural aspects of social networks make a unique contribution to survival, independently of emotional support and the role played in the lives of significant others.

**Conclusion:**

In this elderly Southern European population, the beneficial effects of social networks, social participation, engagement in the life of significant others and having a confidant call for public policies that foster intergenerational and community exchanges.

## Background

The protective effect of social relationships on the mortality of the elderly population has been shown in research using general social network indexes [1-6] or variables related to specific aspects of social relationships [7-22]. Differences among studies in the strength of the associations reported have been related mainly to variations in measurement and analysis strategies. Nevertheless, cultural variability may also explain differences in the associations between social networks and mortality, as shown in a study of three communities in the USA [3]. Research on social relationships and mortality in the Mediterranean region is scant, and most results on this topic are derived from studies conducted in Northern Europe, North America, and East Asia. Studies conducted in different cultures allow for increased variability in the nature and intensity of social contacts which could reveal effects not previously described.

Gender differences in the associations between social relationships and mortality may be culturally dependent, as reflected in conflicting results reported from Finland [8] and other countries [6,15,17,19], and in differential effects observed in three American communities [3]. Socialization depends on gender, and its differential value can translate into dissimilar health effects.

Berkman & Glass [23] have reviewed cumulative evidence on the effect of social networks and support on health and incorporated it into their work. They propose a 'cascade' by which communities' socio-economic and cultural characteristics determine the structural characteristics of social relationships (the social networks). This structure supports functional aspects (psychosocial mechanisms, which include social support) that, in turn, influence health via psychological and physiological pathways. Testing of this model within different cultures is warranted.

The model proposes that social support mediates the effects of social networks. Most work on social support has centred on emotional and instrumental support received by older persons. More pro-active aspects like help provided by the older individual to others and the role he/she plays in their lives have rarely been considered [15,20,21]. Our aim is to test and expand the elements of the Berkman & Glass model among older Mediterranean men and women, including psychosocial aspects related to the social integration of older men and women in the most intimate social circles, comprised of family and friends, as well as their social involvement in their communities.

More specifically, the objectives of this paper are to analyse the influence on survival of social relationships among older men and women and to examine specific psychosocial mechanisms that may explain the effects of social networks on mortality in a Southern European community.

## Methods

The study sample was taken from the first wave (1993) of the cohort study 'Aging in Leganés', whose main purpose was to describe changes in health status, disability and use of services among the community-dwelling elderly, and to relate such changes to social networks and support [24]. Leganés is a town of 173,584 inhabitants in the Madrid (Spain) metropolitan area. A random sample of 1,560 individuals aged 65 years or older was obtained from the municipal roll, stratified by sex and two-year age groups (65–66, 67–68, ...89 or over). Data were collected during two home interviews. During the first visit, trained interviewers asked questions on socioeconomic aspects, health status, Activities of Daily Living (ADLs) and Instrumental Activities of Daily Living (IADLs) disability, depressive symptoms, use of health and social services, and social relationships. During the second visit, physicians inquired about mobility, lifestyle, weight, accidents, vision and hearing ability, and incontinence, and performed a partial physical exam (blood pressure, height, cognitive function, visual acuity, audiometry and mouth inspection). When an individual was unable to answer due to cognitive deficit (Pfeiffer's Short Portable Mental Status Questionnaire score 5 or higher [25]), severe hearing problems, severe illness or aphasia, the interviewer requested the assistance of a proxy respondent to reply to questions unrelated to personal feelings.

### Variables

Vital status for the 1,560 subjects was obtained from the Statistics Office of the Region of Madrid; all deaths through December 31, 1999 were included.

Data on social networks included: *Marital status*; *living arrangements *(alone, only with spouse, with spouse and children, with children without spouse, with others who are not spouse or children); *number of times per month the subject goes to the neighbourhood square (plaza) for a walk or to shop, to social clubs for seniors and to church (or other place of worship); friends contacted (by phone or face to face) at least once in a month; and children and other family members (siblings, nieces/nephews and grandchildren) whom the older person sees or speaks to by phone each month*. Questions on frequency of contacts were adapted from the Yale Health and Aging Project [26].

Based on this information, two indexes were built. The *family ties index *assigns one point for being married, one for having children who the person sees or talks to by phone at least once per month, and one for seeing or speaking by phone to a sibling, niece/nephew or grandchild at least once per month (range 0 to 3). The *social participation index *assigns one point for each of three activities (going to the local square, visiting a seniors' social club or attending church) the individual performs at least once per month (range 0 to 3).

In relation to psychosocial mechanisms, individuals were asked if they had any special person they could share confidences and feelings with, someone they could trust (a *confidant*). In addition, a factor analysis (principal axis factoring, varimax rotation) was carried out on six questions on social support and one question on satisfaction that were asked in relation to each of the individual's ties (spouse, children, other family members, and friends). Information was requested on how frequently the subject: a) felt loved and cared for; b) felt listened to; c) felt criticised; d) helped them; e) felt he/she played an important role in their lives; f) felt useful to them. Possible answers were 'never' (0 points), 'sometimes' (1 point), 'frequently' (2 points), 'always' (3 points). The first two questions were taken from the MacArthur Community Study (USA) [27]. The question on satisfaction with the relationship was scored as follows: 'not satisfied' (0 points), 'slightly satisfied' (1 point), 'somewhat satisfied' (2 points), 'satisfied' (3 points), 'very satisfied' (4 points). Two factors were found (Table 1): Questions a and b and the question regarding satisfaction scored in one factor (except in the analysis for friends, where only questions a and b scored), subsequently named *'received emotional support'*; questions d, e and f scored on a different factor named *'role of the individual in the lives of his/her significant others'*. The items on criticism showed very low communalities and factor loadings on both factors, probably because there were very few 'always' or 'frequently' answers to these questions. KMO indexes were above 0.65. Cronbach's alpha for the eight factors (two per tie) varied between 0.61 and 0.72. For each type of relationship (friends, children, relatives and spouse), two scores were estimated. To reduce the total number of variables in the multivariate models and increase power, the scores for "received emotional support" and "role" were then averaged across relationships.

**Table 1:** Factor loadings, communalities (h^2^), percents of variance explained for principal axis factoring and varimax rotation on the social support and satisfaction items for the four ties.

**Items**	**Children**			**Other family**			**Friends**			**Spouse**		
	Factor 1	Factor 2	h^2^	Factor 1	Factor 2	h^2^	Factor 1	Factor 2	h^2^	Factor 1	Factor 2	h^2^

**Feels loved and cared for**	0.75		0.58		0.69	0.55		0.68	0.48	0.80		0.64
**Feels listened to**	0.69		0.52	0.31	0.51	0.36		0.58	0.36	0.73		0.54
**Feels criticised**			0.06			0.08			0.21			0.08
**Provides help**		0.70	0.49	0.62		0.39	0.55		0.32		0.71	0.52
**Plays an important role**	0.53	0.44	0.48	0.60	0.32	0.46	0.59		0.45	0.32	0.46	0.32
**Feels useful**		0.70	0.54	0.76		0.60	0.82		0.71		0.73	0.53
**Satisfaction**	-0.56		0.33		-0.53	0.32			0.05	-0.56		0.32
**% variance explained by factor and overall variance explained**	40.3	17.3	57.6	39.4	16.5	55.9	31.4	17.6	49	35.7	20.9	56.6

Only factor loadings equal or higher than 0.3 are shown.

The sociodemographic variables collected were *sex*, *age *and *education *(incomplete primary education vs. complete primary education in the multivariate model).

Potential confounders included physical and mental health status, disability and self-rated health. *Physical activity *was categorised as light (sitting down or walking at home); moderate (performing housekeeping tasks, walking outside home); and vigorous (lifting weights, sports). A validated Spanish version of the CES-D [28,29] was used to measure *depressive symptoms*; to avoid the influence of outliers of the skewed distribution of the variable the square root was calculated. A *comorbidity index *was calculated, scoring one for each of the following problems the subject reported: hypertension, cardiac disease, circulation problems, stroke, diabetes, respiratory problems, pain in joints or bones, tumours or cancer, Parkinson's disease, genito-urinary problems and gastrointestinal problems. This index was truncated at 7 in the multivariate analyses to reduce positive skew. *Cognitive status *was ascertained with the *Prueba Cognitiva de Leganés *(PCL), a test developed to screen for cognitive impairment in populations with little education [30,31]. The test produces a score between 0 and 32. Because the distribution was negatively skewed, the square root of 33 minus the score was calculated. For the transformed score, a higher value means more cognitive impairment. *Self-perceived health *was categorized as optimal (very good and good) vs. less than optimal (fair, poor and very poor). Lastly, *disability *was ascertained by asking individuals if they were able to carry out 8 activities of daily living (ADLs) [32] and 8 instrumental activities of daily living (IADLs) [33] alone, with help or not at all. Based on this information, the following categories were created: independent for ADLs and IADLs (able to perform all activities without help); help required for at least one IADL but not for any ADLs; help required for at least one ADL.

### Statistical analysis

The variables were described as percentages, means or medians (in the case of skewed distributions) with their corresponding 95% confidence intervals. For descriptive purposes, results were weighted by the sampling design coefficients. Differences between men and women were tested using chi square, Student-Fisher's t and Mann-Whitney U tests, depending on the indicator selected.

Bivariate analyses used a chi-square test for categorical variables, to test for differences in the percentages of those deceased in each category. For the continuous variables, means or medians for those alive and dead were calculated and the differences tested with a Student-Fisher's t or Mann-Whitney U test.

Multivariate analyses were conducted following a proportional hazards model with non-weighted data. Two models were calculated, one for women and one for men. Variables were introduced using staggered entry according to Glass & Berkman's (social relationships and health) conceptual model. First, sociodemographic and social network variables were introduced; second, psychosocial mechanisms were incorporated; third, lifestyle, disease and disability variables were included as potential confounders. Lastly, a model combining data on men and women was fitted which included only those variables that showed associations in the same direction in both sex-specific models. The statistical significance of interactions of social variables with sex was tested in the combined model.

The proportionality of the model was checked by inspecting the distribution of Schoenfeld residuals against survival time and adding a time-dependent variable to the model [34,35]. The log-linear relationship between the hazard rate and the quantitative independent variables was ascertained by categorizing the quantitative variables in similar range groups to check if the coefficients for these categories followed a linear pattern [35]. Analysis of residuals also enabled us to check this assumption and to detect the existence of influential values in the final model (dfbetas). The presence of co-linearity was examined in accordance with standard criteria [36].

For most variables, missing values were present in less than 5% of the subjects, and for no variable did they exceed 8.5%. In order to minimise the number of individuals lost in multivariate analyses, a modified hot-deck approach was used for imputation. This strategy assigns each individual with a missing value in one variable (receptor) a randomly selected value of the same variable pertaining to a group of subjects (donors) who share certain characteristics with the receptor [37,38].

Statistical analyses were performed using SPSS 11.0 for Windows.

## Results

Of the 1,560 eligible subjects in the sample, 1,283 (82.2%) answered the questionnaire, while 152 (9.7%) refused participation, 120 (7.7%) were absent from home on three occasions and 5 (0.3%) were hospitalized. Of those who answered, 93% underwent the medical exam. Their age, sex, marital status, education and self-perceived health distributions were similar to those of the 1987 Spanish National Health Survey. In 105 cases, information was provided by a proxy due to the subject's cognitive impairment (83.8%), speech or hearing problems (45.2%), severe physical illness (24.2%) or unspecified causes (6.6%). It was possible to register more than one condition for each case. Three individuals did not provide any information on social relationships, and in two cases vital status was not known. This left a final study sample of 1,174 individuals.

In the over 6-year follow-up period, 352 of the 1,174 baseline participants died (30%; 95% CI 27.4–32.7). This rate was lower than that of the 386 eligible subjects not included in the analysis (42.2%; 95% CI 37.3–47.2). Non-response at baseline was higher among women and the very old.

A description of the sample is provided in Table 2. Subjects tended to be married, with little education, and to have health and disability problems (mean co-morbidity was higher than 3, only one-third rated their health as good or very good, and less than half were totally independent). Social relationships were widespread: only 12.5% lived alone; at least half the subjects had monthly contact with all three family ties; the social participation index mean was 1.7 out of a maximum of 3; more than 70% had a confidant and received emotional support; and the score for the role played in the lives of significant others was close to the maximum possible for at least half of the population.

**Table 2:** Distribution of potential risk factors for mortality in the total population and separately by sex

	**Total n = 1328^a^**	**Men n = 574^a ^(43.2%; 95% CI: 40.6–45.9)**	**Women n = 754^a ^(56.8%; 95% CI: 54.1–59.4)**	**p value**
Age^b^	71.5 (71.0–72.0)	70.8 (69.9–71.4)	72.3 (71.6–73.1)	<0.001
**Education**				
Primary completed	21.2 (19.0–23.4)	30.7 (26.9–34.4)	14.0 (11.6–16.5)	<0.001
Primary not completed	63.3 (60.7–65.8)	61.1 (57.2–65.1)	64.9 (61.6–68.4)	
Illiterate	15.5 (13.6–17.5)	8.2 (5.9–10.4)	21.1 (18.2–24.0)	
**Marital status**				
Married	63.7 (61.1–66.3)	85.3 (82.4–88.2)	47.3 (43.7–50.8)	<0.001
Single	3.6 (2.5–4.5)	1.4 (0.4–2.3)	5.2 (3.6–6.7)	
Widowed or separated	32.7 (30.2–35.3)	13.3 (10.5–16.0)	47.5 (44.0–51.1)	
**Living arrangements**				
Alone	12.5 (10.7–14.3)	5.6 (3.7–7.5)	17.8 (15.0–20.5)	<0.001
Only with spouse	42.7 (40.0–45.4)	56.4 (52.2–60.3)	32.4 (29.0–35.7)	
With children without spouse	20.5 (18.3–22.7)	8.2 (5.9–10.4)	29.8 (26.6–33.1)	
With spouse and children	20.3 (18.2–22.5)	27.9 (24.2–31.5)	14.6 (12.1–17.1)	
With others who are not the spouse or children	3.9 (2.9–5.0)	1.9 (0.8–3.0)	5.4 (3.8–7.1)	
**Family ties index (0–3)^b^**	3 (3-3)	3 (3-3)	2 (2-2)	<0.001
**Social participation index (0–3)^c^**	1.7 (1.6–1.7)	1.8 (1.7–1.9)	1.6 (1.6–1.7)	<0.001
**Contacts with friends**	48.0 (45.3–50.7)	55.7 (51.7–59.8)	42.0 (38.5–45.6)	<0.001
**Confidant**	70.6 (68.2–73.1)	70.2 (66.5–74.0)	71.0 (67.7–74.2)	0.77
**Average received emotional support (0–3,5)^b^**	2.9 (2.9–2.9)	2.9 (2.9–3.0)	2.9 (2.8–3.0)	0.20
**Average role in significant others' lives (0–3)^b^**	2.2 (2.2-2.2)	2.3 (2.2–2.3)	2.2 (2.1–2.2)	0.01
**Physical activity**				
Light	24.3 (22.0–26.6)	16.6 (13.5–19.6)	30.2 (27.0–33.5)	<0.001
Moderate	72.7 (70.3–75.1)	79.6 (76.3–82.9)	67.4 (64.0–70.7)	
Vigorous	3.0 (2.1–3.9)	3.8 (2.3–5.4)	2.4 (1.3–3.5)	
**Depressive symptoms (0–60)^b^**	9 (8–9)	6 (6–7)	12 (11–13)	<0.001
**Comorbidity (0–10)^c^**	3.3 (3.2–3.4)	2.8 (2.7–2.9)	3.7 (3.6–3.8)	
***Prueba Cognitiva de Leganés *(Cognitive Testing in Leganés) (0–32)^b,d^**	26 (26-26)	26 (26-26)	26 (25–26)	0.03
**Self-perceived health**				
Very good/good	31.7 (29.2–34.2)	41.2 (37.3–45.3)	24.4 (21.3–27.5)	<0.001
Average	50.1 (47.5–52.8)	47.0 (43.0–51.1)	52.5 (49.0–56.1)	
Poor/very poor	18.2 (16.1–20.2)	11.8 (9.2–14.5)	23.1 (20.1–26.1)	
**Disability**				
Independent for ADLs and IADLs	48.1 (45.4–50.7)	58.5 (54.5–62.6)	40.1 (36.6–43.6)	<0.001
Not independent for IADLs	30.8 (28.3–33.3)	28.6 (24.9–32.3)	32.5 (29.2–35.8)	
Not independent for ADLs	21.2 (19.0–23.4)	12.9 (10.2–15.6)	27.5 (24.3–30.6)	

^a^Weighted results. ^b^Median and its 95% confidence interval. ^c^Mean and its 95% confidence interval. ^d^Not transformed data (a higher score means a better cognitive function). CI: Confidence interval. ADLs: Activities of Daily Living. IADLs: Instrumental Activities of Daily Living.

Gender differences were observed with respect to most variables: women were older, less educated, in worse health, with more depressive symptoms and more disability. Women exercised less than men, they lived alone more often, were more frequently widowed, and their family ties index was lower. In addition, their social participation activities were less diversified. Women contacted friends less frequently, and their appraisal of their role in the lives of their significant others was lower as compared to men. Having a confidant was as frequent among men as among women.

Bivariate analyses showed a significant association between a higher level of social relationships and survival (data not shown). Only two variables, receipt of emotional support (in both sexes) and contacts with friends were not statistically associated with survival. Potential confounders were related to mortality in the expected direction except for co-morbidity (in women).

Tables 3 and 4 shows the models for the association between social relationships and survival separately for women and men, respectively, while Table 5 shows the same analysis for both sexes combined. No interaction of social network variables by sex was significant. Since marital status is a component of the family ties index and living arrangements are highly correlated with marital status, we have excluded both variables from the analysis. Receipt of emotional support was not included in the combined model because it showed non-significant, opposite associations for each sex, rendering the calculation of an across-sexes effect meaningless.

**Table 3:** Mortality Hazard Rate Ratios for social relationships, adjusted for health and disability in women

**WOMEN (n = 573; deceased = 138)^a^**	**Network model**	**Psychosocial mechanisms model**	**Complete model**
**Age**	**1.12 (1.09–1.15)**	**1.11 (1.08–1.15)**	**1.10 (1.07–1.14)**
**Education:**			
Primary completed	1	1	1
Primary not completed	1.23 (0.72–2.11)	1.22 (0.71–2.10)	1.12 (0.64–1.95)
**Family ties index:**			
< 3	1	1	1
3	0.90 (0.58–1.42)	0.91 (0.58–1.42)	0.87 (0.55–1.37)
**Friends contacted:**			
No	1	1	1
Yes	0.95 (0.65–1.39)	0.97 (0.66–1.43)	1.06 (0.71–1.57)
**Social participation index**	**0.78 (0.64–0.96)**	0.82 (0.67–1.00)	0.90 (0.73–1.12)
**Confidant:**			
No/does not know		1	1
Yes		0.80 (0.56–1.14)	0.79 (0.55–1.13)
**Received emotional support**		1.12 (0.79–1.59)	1.20 (0.84–1.71)
**Role in significant others' lives**		0.78 (0.60–1.01)	0.87 (0.66–1.14)
**Physical activity:**			
Moderate/vigorous			1
Light			1.21 (0.79–1.86)
**Co-morbidity**			1.00 (0.90–1.12)
**Square root of depressive symptoms**			1.09 (0.95–1.24)
**Cognitive score**			1.10 (0.90–1.35)
**Self-perceived health:**			
Very good/good			1
Average/poor/very poor			1.20 (0.74–1.95)
**Disability:**			
Independent			1
Not independent for IADLs			1.18 (0.65–2.13)
Not independent for ADLs			1.55 (0.82–2.93)

^a^Non-weighted results. Bold: indicates results significant at a 95% level. ADLs: Activities of daily living. IADLs: Instrumental activities of daily living.

**Table 4:** Mortality Hazard Rate Ratios for social relationships, adjusted for health and disability in men

**MEN (n = 601; deceased = 214)^a^**	**Network model**	**Psychosocial mechanisms model**	**Complete model**
**Age**	**1.08 (1.06–1.10)**	**1.08 (1.06–1.10)**	**1.07 (1.04–1.09)**
**Education:**			
Primary completed	1	1	1
Primary not completed	1.15 (0.84–1.57)	1.16 (0.85–1.58)	0.97 (0.70–1.34)
**Family ties index:**			
< 3	1	1	1
3	**0.68 (0.51–0.91)**	0.77 (0.57–1.03)	0.75 (0.56–1.01)
**Friends contacted:**			
No	1	1	1
Yes	0.99 (0.75–1.30)	1.10 (0.83–1.47)	1.16 (0.87–1.55)
**Social participation index**	**0.83 (0.71–0.97)**	**0.84 (0.72–0.98)**	0.89 (0.76–1.04)
**Confidant:**			
No/does not know		1	1
Yes		**0.70 (0.52–0.93)**	0.77 (0.57–1.04)
**Received emotional support**		0.82 (0.59–1.13)	0.79 (0.57–1.10)
**Role in significant others' lives**		0.81 (0.65–1.03)	0.87 (0.69–1.10)
**Physical activity:**			
Moderate/vigorous			1
Light			**1.50 (1.06–2.13)**
**Co-morbidity**			1.05 (0.97–1.14)
**Square root of depressive symptoms**			1.03 (0.92–1.15)
**Cognitive score**			**1.25 (1.08–1.45)**
**Self-perceived health:**			
Very good/good			1
Average/poor/very poor			1.37 (0.98–1.92)
**Disability:**			
Independent			1
Not independent for IADLs			1.22 (0.85–1.76)
Not independent for ADLs			0.93 (0.59–1.47)

^a^Non-weighted results. Bold: indicates results significant at a 95% level. ADLs: Activities of daily living. IADLs: Instrumental activities of daily living.

**Table 5:** Mortality Hazard Rates Ratios for social relationships adjusted for health and disability, in men and women combined

**ALL (n = 1174; deceased = 352)^a^**	**Network model**	**Psychosocial mechanisms model**	**Complete model**
**Sex:**			
Women	1	1	1
Men	**1.92 (1.50–2.46)**	**1.92 (1.50–2.46)**	**2.20 (1.71–2.84)**
**Age**	**1.10 (1.08–1.11)**	**1.09 (1.07–1.11)**	**1.08 (1.06–1.10)**
**Education:**			
Primary completed	1	1	1
Primary not completed	1.16 (0.88–1.51)	1.18 (0.90–1.54)	1.01 (0.77–1.34)
**Family ties index:**			
< 3	1	1	1
3	**0.73 (0.57–0.93)**	0.79 (0.62–1.01)	**0.77 (0.60–0.98)**
**Friends contacted:**			
No	1	1	1
Yes	0.97 (0.78–1.21)	1.05 (0.84–1.32)	1.12 (0.89–1.41)
**Social participation index**	**0.80 (0.71–0.91)**	**0.82 (0.73–0.93)**	0.88 (0.78–1.00)
**Confidant:**			
No/does not know		1	1
Yes		**0.72 (0.58–0.90)**	**0.75 (0.60–0.95)**
**Role in significant others' lives**		**0.79 (0.67–0.93)**	0.86 (0.73–1.02)
**Physical activity:**			
Moderate/vigorous			1
Light			**1.38 (1.06–1.79)**
**Co-morbidity**			1.03 (0.97–1.10)
**Square root of depressive symptoms**			1.04 (0.96–1.13)
**Cognitive score**			**1.20 (1.07–1.35)**
**Self-perceived health:**			
Very good/good			1
Average/poor/very poor			**1.32 (1.01–1.74)**
**Disability:**			
Independent			1
Not independent for IADLs			1.17 (0.86–1.59)
Not independent for ADLs			1.10 (0.77–1.58)

^a^Non-weighted results. Bold: indicates results significant at a 95% level. ADLs: Activities of daily living. IADLs: Instrumental activities of daily living.

The family ties index was dichotomised because there were too few individuals with one or no family ties to allow for estimation of coefficients in these categories. These individuals were merged with those having an average of two monthly contacts. Redefined in this fashion, this variable was very significant for men, and its effect was partially explained by health and disability variables.

**Figure 1:**
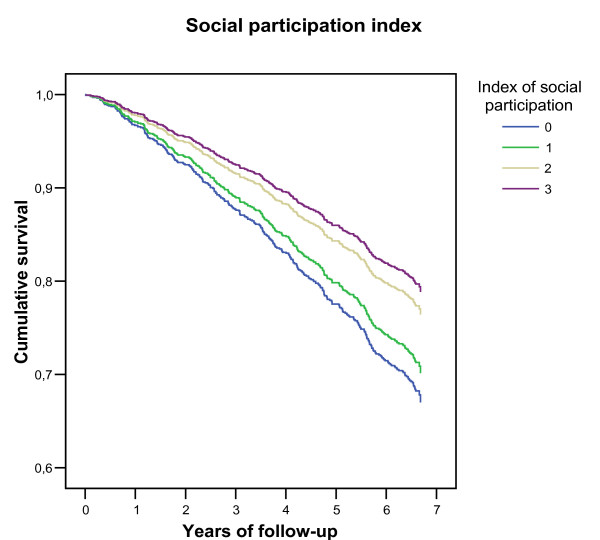
Estimated survival probabilities of individuals according to the number of social activities they take part in (social participation index), after adjusting for sociodemographic and social relationships variables.

Contacts with friends had no effect on survival. Diversified social participation had a protective effect, and showed a similar gradient for men and women. However, the association lost significance when all health and disability variables were included in the model. Figure 1 displays the probability of being alive at each follow-up year for each more activity the individuals took part in, after adjustment for sociodemographic and other social relationships variables (when health and disability variables were included, the gradient was partially lost, with the hazard ratio for those performing one activity closely approaching that of persons who performed no activities). After adjusting for socio-demographic, other social relationships and health and disability variables, the risk of death of an individual who did not participate in any of the activities was 1.5 times (95% CI 1.3–1.7) the risk of a full participant.

Having a confidant showed a greater protective effect in men, but sex differences disappeared when health and disability variables were included. When men and women were combined, the relationship was significant even with all variables in the model: having a confidant is associated with a 25% (95% CI 5–40%) reduction in the mortality risk

Receipt of emotional support was not associated with mortality. An increasing role of the individual in the lives of significant others showed a protective effect in both sexes, which reached statistical significance only in the combined model. The significance was lost after adjusting for health and disability. Again, no evidence against a gradient of the variable effect was found.

## Discussion

Four components of social relationships are associated with 6-year survival in this elderly Southern European population: contacts with family ties, participation in social activities, having a confidant, and playing a meaningful role in the lives of significant others. Contacts with family ties and having a confidant remain significantly associated with improved survival, whereas participation in social activities and meaningful role in the lives of significant others lose significance after adjustment for health and disability variables. This suggests that the effects of social relationships on survival may be partly mediated by the fostering of better health and function.

This population enjoys relatively high levels of social contact, centred on family life, as would be expected in a Mediterranean community. Nevertheless, these levels are not as high for women as for men. A National Survey has confirmed this finding, reporting fewer contacts with friends and relatives among Spanish women [39]. Women in our cohort show worse family ties indexes mainly because they are older and, consequently, more frequently widowed. In addition, they have fewer contacts with friends and less participation in social life. This last result is congruent with findings in Finland [8] and Taiwan [19], but dissimilar to those in France [6] and Denmark [15] (where there are no differences between sexes) and the USA [11] (where women enjoy a larger social network and more social participation). Our tentative explanation for these differences is that women in our cohort have lived most of their lives in a cultural background that favoured their engagement in family life over their participation in activities outside home. We can hypothesize also that the still predominant patriarchal way of thinking in the Mediterranean culture has driven downward these women's self-perception of their capacity of helping and influencing others' lives.

Our analyses show similar protective associations between social relationships and mortality for both sexes (apart from that of the family ties index), so the more limited social relationships women enjoy make them less able to benefit from them. Nevertheless, it is worth mentioning that one of the variables most strongly associated with survival – having a confidant – is equally distributed among men and women. Differences by sex in the association between community participation and survival in the elderly were not observed in another European community [8], but they were present in a Taiwanese one [19]. A USA study that included elderly and young individuals and used a general index of social integration described differential effects between the sexes [3]. It is hard to say whether or not these conflicting results can be attributed to the use of differential scales or to real geographical-cultural effects.

Men with the highest diversity of family ties enjoy better survival. Out of the 163 men with fewer than three family ties, 128 were widowers, while only 36 had no children and 30 had no close relatives. Moreover, when this variable was replaced in the model by marital status (data not shown), the protective effect of being married was very similar to that of the dichotomised family ties index. Therefore, what our dichotomised index is measuring is probably the beneficial effect of being married for men, a finding well reported in the literature on the elderly [6,7].

Community activities (social participation) have generally been reported as predictors of survival [2,8-10,18], although there is conflicting evidence from Sweden [40]. This is not due to higher physical and functional levels of those who participate in them, because, in our study and others, adjustment for health and function does not change the magnitude of the association of community participation with mortality. Moreover, the same effects have been reported in elderly people with different levels of physical activity [41]. In the seminal Tecumseh study [42], leisure activities that did not include social contact (reading, watching TV) did not confer the protective effect generated by social activities, which rules out attributing the beneficial associations observed to entertainment alone. Qualitative research in Spain [43] sheds some light on the positive effects of community activities on mortality: For elderly people, participation in these activities is a way to maintain full participation in society and show that being old does not equal being useless, passive and dependent. In addition, social activities provide room for personal contacts and a daily routine that is a substitute for productive and reproductive work.

Having a confidant appears to be a very strong predictor of survival. It is somewhat surprising that this feature has not been thoroughly investigated in previous studies dealing with mortality. We are aware of only two pieces of research [22,44] which included this variable: no effect was observed in the last one, although its small sample size may have limited power. On the contrary, the Australian cohort study found significant protective effects for having a confidant. The mechanisms through which having this kind of support influence survival are included in the formulation of the question we used to explore this topic. Our item defined a confidant as someone whom the subject can talk to, confide in and trust. Therefore, it behaves as a summary measure of various health-related psychosocial mechanisms. Apart from this increase in overall survival, confidant availability has been reported to reduce the risk of cardiac events after infarction [45], cardiovascular death in patients with ischemic disease [46] and depressive symptomatology incidence among the elderly [47].

The Australian study [22] and others [14,19] have found that having and contacting friends postpones death. Our results show this not to be the case in a Mediterranean community where, probably, family ties are more highly regarded. Family ties appear to be more important for survival, as shown by the protective effect of the family ties index in men, and the fact that, in our cohort, 90% of women's and 98% of men's confidants were members of their families.

Of the other two psychosocial mechanisms considered, receipt of social support and playing an important role in the lives of significant others, only the latter is protective. A study in Japan found that a sense of "present usefulness to others and society" in elderly participants was predictive of longer survival even after adjusting for self-rated health [21]. However, the sense of usefulness was evaluated with a single question, whereas we used a scale, a procedure more suitable for measuring elaborated constructs. Conceptually related variables, such as the ability to take care of others among functionally impaired elderly persons [15] or the emotional and instrumental support given to the spouse among the community dwelling elderly [20] have shown some beneficial effects. Ostir, Simonsick, Kasper & Guralnik [48] measured the rate of satisfaction of elderly disabled women with respect to the help they provided to family, friends and community organizations. They found that higher levels were associated protectively with better lower-body function as well as less ADL difficulty, hospitalisation and mortality, even after adjusting for socio-demographic, medical conditions and baseline disability variables. The finding that survival is associated with what is given by the individual (his/her role) and not with what is received (emotional support) can be attributed to the fact that in the first case the subject plays an active role that can provide purpose or meaning to life and helps him/her remain mentally and physically active. In a recent study, feelings of worth and emotional support were also associated with survival in very elderly women [49].

This paper provides limited information on the pathways through which social networks generate their effects. The introduction of psychosocial mechanisms does not give rise to a major change in the association between mortality and social network variables; therefore the structural components of social relationships generate an effect on health that is not fully explained by the functions considered in the model – a result at odds with the scheme proposed by Berkman & Glass.

The question remains as to the extent to which our results can be applied to other populations. The elderly of Leganés have generally moved to Madrid from various central regions of Spain, and their age, sex, marital status, education and self-perceived health distributions represent that of elderly Spaniards as a whole. Nevertheless, cultural differences among the Spanish regions exist, although in all of them, as in other Southern European communities, family plays a central role in the social networks of the older population. Our results are based on a Mediterranean community. Since social relationship patterns are culturally dependent, it would be necessary to test our findings on the effects of roles and confidants in other populations.

### Methodological considerations

The aim of *Aging in Leganés *was to analyse the impact of social relationships on health, function and use of services. Therefore, the information collected on this topic is very detailed. This applies especially to psychosocial mechanisms – an area which has been studied less often. These complex constructs require batteries of questions to allow collection of valid information [50]. For this study, a new scale was developed based on previous research to separate two types of social support in relation to each tie: receipt of emotional support and an evaluation of the role the individual plays in the lives of significant others. To our knowledge, our role scale is the first to deal with this concept in survival analyses.

The limited evidence available indicates that contacts with different types of ties produce differential effects [14]. We attempted to study the effect of each tie separately, but due to sample size limitations, we were forced to use summary indexes across all relationships.

Although not suitable for relationship-specific analyses, the 6-year follow-up has provided a number of deaths large enough to achieve reasonable power. The baseline survey had a high response rate, and the ascertainment of vital status was virtually complete.

## Conclusion

The evidence provided by numerous observational studies on the protective effects of social relationships on mortality is solid enough to develop and evaluate interventions which promote social networks and support of the elderly population. Nevertheless, interventions should not be directed only at the individual level. As Berkman & Glass reflect in their conceptual scheme, social relationships are determined by sociodemographic, cultural and political forces. Positive effects on survival and quality of life may be produced by public policies that regulate labour markets and taxation; allow for conciliation of work and care of family members; promote the acquisition of larger dwellings or dwellings closer to the family's usual place of residence; facilitate access to home care for the elderly who wish to stay at home as long as possible; and empower civil society so that spaces for community intergenerational interaction can flourish.

## Competing interests

The author(s) declare that they have no competing interests.

## Authors' contributions

ARL conceived the study, performed the statistical analysis and drafted the manuscript. MVZ and AO contributed with intellectual discussions and comments on drafts and with the critical revision of the final version. AO is the current principal investigator of the cohort study "Aging in Leganés". All authors participated in the design of the study and read and approved the final manuscript.

## Pre-publication history

The pre-publication history for this paper can be accessed here:


